# Inverse relation between FASN expression in human adipose tissue and the insulin resistance level

**DOI:** 10.1186/1743-7075-7-3

**Published:** 2010-01-15

**Authors:** María D Mayas, Francisco J Ortega, Manuel Macías-González, Rosa Bernal, Ricardo Gómez-Huelgas, José M Fernández-Real, Francisco J Tinahones

**Affiliations:** 1 Servicio de Endocrinología y Nutrición, Hospital Clínico Universitario Virgen de Victoria de Málaga, España; 2 CIBEROBN (CB06/03/010), Instituto de Salud Carlos III, España; 3 Servicio de Diabetes, Endocrinología y Nutrición, Instituto de Investigación Biomédica de Girona, España; 4 Laboratorio de Investigación, Fundación IMABIS, Málaga, España; 5 Servicio de Medicina Interna, Hospital Universitario Carlos Haya de Málaga, España

## Abstract

**Background:**

Adipose tissue is a key regulator of energy balance playing an active role in lipid storage and may be a dynamic buffer to control fatty acid flux. Just like PPARγ, fatty acid synthesis enzymes such as FASN have been implicated in almost all aspects of human metabolic alterations such as obesity, insulin resistance or dyslipemia. The aim of this work is to investigate how FASN and PPARγ expression in human adipose tissue is related to carbohydrate metabolism dysfunction and obesity.

**Methods:**

The study included eighty-seven patients which were classified according to their BMI and to their glycaemia levels in order to study FASN and PPARγ gene expression levels, anthropometric and biochemical variables.

**Results:**

The main result of this work is the close relation between FASN expression level and the factors that lead to hyperglycemic state (increased values of glucose levels, HOMA-IR, HbA1c, BMI and triglycerides). The correlation of the enzyme with these parameters is inversely proportional. On the other hand, PPARγ is not related to carbohydrate metabolism.

**Conclusions:**

We can demonstrate that FASN expression is a good candidate to study the pathophysiology of type II diabetes and obesity in humans.

## Background

Adipose tissue is recognized as a key regulator of energy balance, playing an active role in lipid storage with multiple distinct deposits (subcutaneous, intra-abdominal and intrathoracic) [1]. Indeed, adipocytes of visceral abdominal fat origin are more endocrinologically active than the subcutaneous variety [2]. In addition, adipose tissue can buffer, synthesize and secrete a wide range of endocrinal products into circulating blood that is influential on the systemic metabolism and may be directly involved in the pathogenesis of associated complications such as obesity, diabetes, vascular damage and atherosclerosis [1,3]. Thus, adipose tissue may serve as a dynamic buffer to control fatty acid (FA) flux in response to changing energy demands: in the fasting state, adipose tissue releases FAs, whereas in the fed state, adipocytes change to "absorb" FAs from the circulation, mainly from circulating triglycerides (TG) [4,5]. This function is known to be altered in obese subjects with metabolic syndrome features (insulin resistance, obesity, dyslipemia, inflammation, atherosclerosis and hypertension) [6,7].

The nuclear receptor peroxisome proliferator-activated receptor gamma (PPARγ) is a ligand-activated transcription factor, member of the nuclear hormone receptor superfamily, which functions as a heterodimer with a retinoid X receptor (RXR) [8]. The actions of PPARγ are mediated by two protein isoforms which are derived from the same gene by alternative promoter usage and splicing: the widely expressed PPARγ1 and the adipose tissue-restricted PPARγ2 [9]. The activation of PPARγ leads to adipocyte differentiation and fatty-acid storage, whereas it represses genes that induce lipolysis and the release of free fatty acids (FFAs) in adipocytes [10]. Authors have shown that the loss-of-function mutation of PPARγ results in severe insulin resistance and causes elevated TG and decreased high density lipoprotein-cholesterol levels in humans while increased PPARγ activity enhances insulin sensitivity and improves dyslipidemia in insulin-resistant individuals [11].

PPARγ transcriptionally regulates many genes involved in metabolism [12], even those involved in the synthesis of FAs. There are two sources of FA, exogenously-derived (dietary) and endogenously-synthesized FA, both are essential constituents of biological membrane lipids and important substrates for energy metabolism. The biosynthesis of the latter is catalysed by Fatty Acid Synthase (FASN) and Acetyl-CoA Carboxylase (ACC), key enzymes of lipogenesis that may play a crucial role in the weight variability of abdominal adipose tissue [13]. Specifically, FASN (EC 2.3.1.85) is a multifunctional enzymatic complex, important in the regulation of body weight and the development of obesity [13-15] and necessary for de novo synthesis of long-chain saturated FAs from acetyl coenzyme A (CoA), malonyl-CoA and NADPH. The expression of this enzyme is highly dependent on nutritional conditions in lipogenic tissues. FASN-catalysed endogenous FA biosynthesis in liver and adipose tissue is stimulated by a high carbohydrate diet, whereas it is suppressed by the presence of small amounts of FA in the diet and by fasting [16].

There are several studies that connect FASN activity/expression with metabolic alterations in humans such as obesity, dyslipemia, insulin resistance and altered adipocytokine serum profile [17]. Although there are authors that have shown how FASN gene expression is significantly higher in obese vs lean individuals [17-19], there are studies that found the way in which FASN mRNA expression was decreased in the subcutaneous adipose tissue of obese vs lean individuals [20]. Divergent findings may be explained by differences in metabolic parameters and the size of the study population. We contribute to study the role of FASN with a general population with a wide range of body mass index (BMI) and metabolic parameters, in order to clarify the association between FASN activity/expression, the grade of insulin resistance and obesity-related insulin resistance.

## Methods

### Experimental subjects

The study included 87 healthy persons (35 men and 52 women) who underwent laparoscopic surgery procedures (hiatus hernia repair or cholecystectomies). Patients were classified into three groups according to BMI: normal (BMI < 25), overweight (25 ≤ BMI < 30) and obese (BMI ≥ 30). Patients were also classified into normoglycemic (no diabetes antecedents and glucose levels in a fast state ≤ 110 mg/dl) and hyperglycemic (diabetics or people with basal glycaemia values in a fast state >110 mg/dl) groups. This study was approved by the Hospital's Ethical Committee and all participants signed their consent after being fully informed of its goal and characteristics.

### Study design

Before surgery and after an overnight fast, the patient's height and weight was measured to calculate the BMI and the waist and circumference to calculate the waist to hip ratio (W-H). In addition, systolic blood pressure (SBP) and diastolic blood pressure (DBP) were noted. During surgical intervention, biopsies of visceral adipose tissue were immediately frozen in liquid nitrogen and stored at -80°C for gene expression analysis. Blood samples were collected; serum and plasma were separated in aliquots within 30 min of extraction, and immediately frozen at -80°C.

Biochemical variables were: glucose, cholesterol, TG, high density lipoprotein-cholesterol (HDL-c) and low density lipoprotein-cholesterol (LDL-c), glycated haemoglobin (HbA1c), C-reactive protein (CRP) and all were measured in a Dimension Autoanalyzer (Dade Behring, Deerfield, IL) in duplicate. Serum insulin concentration was analyzed by an immunoradiometric assay (IRMA) (BioSource International, Camarillo, CA). Leptin and adiponectin were analysed by enzyme immunoassay (ELISA) kits (Mediagnost, Reutlingen, Germany and DRG Diagnostics GmbH, Germany, respectively). The homeostasis model assessment of insulin resistance (HOMA-IR) was calculated as follows: fasting glucose (mg/dl) * fasting insulin (uU/ml)/405 [21].

RNA extraction and real time quantitative PCR: Adipose tissue RNA isolation was performed by homogenization with an ULTRATURRAX T25 basic (IKA Werke GmbH, Staufen, Germany) using Trizol reagent (Invitrogen, Barcelona, Spain). Samples were purified using RNAEasy Mini kit (QIAGEN, Barcelona, Spain) and treated with DNase (RNase-free DNase Set, Qiagen). For first strand cDNA synthesis, constant amounts of 1 μg of total RNA were reverse transcribed using random hexamers as primers and Transcriptor Reverse Transcriptase (Roche, Mannheim, Germany). Gene expression was assessed by real time PCR using an ABI Prism 7000 Sequence Detection System (*Applied Biosystems, Darmstadt, Germany*), using TaqMan^® ^technology suitable for relative genetic FASN expression quantification. The reaction was performed, following the manufacturers protocol, in a final volume of 25 μl. The cycle program consisted of an initial denaturing of 10 min at 95°C, followed by 40 15 sec denaturizing phase cycles at 95°C and a 1 min annealing and extension phase at 60°C. Commercially available and pre-validated TaqMan^® ^primer/probe sets were used as follows: PPIA (*4333763, RefSeq. NM_002046.3, Cyclophilin A *(PPIA), used as endogenous control for the target gene in each reaction) and FASN (*Hs00188012_m1, RefSeq. NM_004104.4, Fatty Acid Synthase*). A threshold cycle (Ct value) was obtained for each amplification curve and a ΔCt value was first calculated by subtracting the Ct value for human PPIA cDNA from the Ct value for each sample and transcript. Fold changes compared with the endogenous control were then determined by calculating 2^-ΔCt^, so FASN expression results are expressed as the expression ratio relative to PPIA gene expression according to the manufacturer's guidelines. The transcript levels of nuclear receptors PPARγ1 and PPARγ2 were quantified by real-time reverse transcription RT-PCR, using *LightCycler*^® ^technology (Roche Diagnostic, Rotkreuz, Switzerland) with SYBR Green detection. The primers for the PCR reaction (Sigma Proligo) were: a common reverse primer for PPARγ1 and for PPARγ2, CTTCCATTACCGAGAGATCC. The forward primer for PPARγ1 was AAAGAAGGCGACAACTAAACC and GCGATTCCTTCACTGATAC for PPARγ2. A standard curve was created with serial dilutions of a PCR fragment from human adipose tissue total RNA (Clontech Laboratories, Inc., Mountain View, CA). For quantification purposes, PPARγ mRNA levels were always reported to the levels of β-actin, constitutively expressed gene. Primers for β-actin were AACTGGAACGGTGAAGGTGAC as forward and TGTGGACTTGGGAGAGGACTG as reverse. All samples were quantified in duplicate and positive and negative controls were included in all the reactions.

### Statistical analysis

Data are expressed as mean ± standard deviation (SD). The differences in the study variables of normal, overweight and obese individuals were compared with an ANOVA or Student test for independent samples. Pearson's correlation coefficients were calculated to estimate the linear correlations between variables and the confidence interval was of 95%. Multiple regression analysis was used to study which variables were associated with FASN expression levels. Values were considered to be statistically significant when P ≤ 0.05. The statistical analyses and graphics were performed using the program SPSS (Version 11.5 for Windows; SPSS, Chicago; IL).

## Results

The anthropometric and biochemical variables of the studied subjects and FASN and PPARγ gene expression of the three groups (normal, overweight and obese) are summarized in Table 1. BMI is directly related to SBP values (P < 0.01), W-H ratio (P < 0.05), glucose (P < 0.01), HbA1c (P < 0.01), HOMA-IR (P < 0.01), leptin (P < 0.01) and TG (P < 0.01) levels and inversely related to FASN expression (P < 0.01) and adiponectin levels (P < 0.01).

**Table 1:** Anthropometrical and biochemical characteristics of study subjects: normal, overweight and obese individuals

		Means	SD	P			Means	SD	P
BMI	Normal	22.453	2.317	0.00	TG	Normal	83.353	32.017	0.00
	Overweight	27.389	1.563			Overweight	141.600	81.945	
	Obese	34.393	3.801			Obese	141.286	63.364	
	Total	26.554	4.856			Total	117.687	68.273	

SBP	Normal	121.543	20.860	0.00	LDL-c	Normal	123.294	28.510	0.88
	Overweight	131.114	15.854			Overweight	122.229	28.823	
	Obese	140.857	17.110			Obese	126.857	26.921	
	Total	128.750	19.392			Total	123.446	28.091	

DBP	Normal	75.086	12.344	0.25	HDL-c	Normal	55.853	15.182	0.30
	Overweight	76.857	10.097			Overweight	51.800	12.211	
	Obese	80.714	6.390			Obese	50.286	9.659	
	Total	76.762	10.696			Total	53.205	13.213	

W-H ratio	Normal	0.862	0.080	0.02	CRP	Normal	5.285	17.498	0.16
	Overweight	0.897	0.068			Overweight	3.254	2.576	
	Obese	0.930	0.093			Obese	11.143	14.854	
	Total	0.888	0.081			Total	5.417	12.983	

Insulin	Normal	10.824	6.162	0.11	Adiponectin	Normal	23.423	14.405	0.00
	Overweight	13.537	6.134			Overweight	13.794	5.904	
	Obese	14.736	7.938			Obese	16.963	6.891	
	Total	12.685	6.593			Total	18.190	11.087	

Glycaemia	Normal	79.735	8.972	0.01	Leptin	Normal	12.005	14.011	0.00
	Overweight	100.200	37.802			Overweight	18.745	16.660	
	Obese	93.857	11.455			Obese	33.039	22.406	
	Total	90.747	27.125			Total	18.730	18.300	

HbA1c	Normal	5.509	0.330	0.00	FASN	Normal	0.316	0.179	0.00
	Overweight	5.829	0.688			Overweight	0.194	0.151	
	Obese	6.064	0.472			Obese	0.127	0.100	
	Total	5.737	0.565			Total	0.237	0.172	

HOMA-IR	Normal	2.129	1.103	0.00	PPARγ1	Normal	0.031	0.048	0.39
	Overweight	3.373	1.911			Overweight	0.086	0.232	
	Obese	3.639	2.201			Obese	0.100	0.267	
	Total	2.914	1.787			Total	0.063	0.181	

Cholesterol	Normal	199.529	39.368	0.96	PPARγ2	Normal	0.006	0.004	0.36
	Overweight	200.543	36.575			Overweight	0.007	0.005	
	Obese	202.786	33.971			Obese	0.005	0.003	
	Total	200.506	36.914			Total	0.007	0.004	

Values are presented as means ± SD. BMI, body mass index (Kg/m^2^); SBP, systolic blood pressure (mm Hg); DBP, diastolic blood pressure (mm Hg); W-H ratio, waist to hip ratio; Insulin (UI/ml); Glycaemia (mg/dl); HbA1c, glycated haemoglobin (%); HOMA-IR, homeostasis model assessment ((gluc mg/dl*insul U/ml)/405); Cholesterol (mg/dl); TG, triglycerides (mg/dl); LDL-c, low density lipoprotein-cholesterol (mg/dl); HDL-c, high density lipoprotein-cholesterol (mg/dl); CRP, c-reactive protein (mg/l); Adiponectin (ng/ml); Leptin (ng/ml); FASN, fatty acid synthase; PPARγ, peroxisome proliferator-activated receptor.

Comparisons between normoglycemic and hyperglycemic subjects (Table 2) have shown that the last group had significantly higher baseline TG readings (P < 0.05), BMI (P < 0.05), glucose (P < 0.01), HbA1c (P < 0.01) and HOMA-IR (P < 0.01), and lower levels of FASN expression (P < 0.01). No significant changes were detected in the other variables.

**Table 2:** Anthropometrical and biochemical characteristics of study subjects: with and without high glycaemia

Patients	Control	High Glycaemia	P
BMI	26.18 ± 4.80	29.51 ± 4.29	0.03
SBP	126.70 ± 18.79	137.75 ± 16.38	0.06
DBP	76.35 ± 10.13	77.50 ± 11.68	0.72
W-H	0.89 ± 0.08	0.91 ± 0.07	0.26
Insulin	12.28 ± 6.29	14.93 ± 8.02	0.20
Glucose	83.78 ± 9.40	136.36 ± 52.61	0.01
HbA1c	5.59 ± 0.38	6.70 ± 0.66	0.00
HOMA-IR	2.56 ± 1.38	5.02 ± 2.50	0.01
Cholesterol	201.43 ± 34.37	194.45 ± 52.35	0.68
TG	105.78 ± 49.68	195.64 ± 113.98	0.03
LDL-c	125.36 ± 26.61	110.91 ± 35.28	0.11
HDL-c	53.81 ± 13.35	49.27 ± 12.10	0.29
CRP	5.75 ± 13.89	3.23 ± 2.58	0.55
Adiponectin	18.94 ± 11.68	14.08 ± 5.68	0.16
Leptin	17.32 ± 17.77	26.83 ± 19.96	0.10
FASN	0.27 ± 0.17	0.08 ± 0.03	0.00
PPARγ1	0.07 ± 0.20	0.03 ± 0.03	0.49
PPARγ2	0.01 ± 0.00	0.01 ± 0.01	0.98

Values are presented as means ± SD. BMI, body mass index (Kg/m^2^); SBP, systolic blood pressure (mm Hg); DBP, diastolic blood pressure (mm Hg); W-H ratio, waist to hip ratio; Insulin (UI/ml); Glycaemia (mg/dl); HbA1c, glycated haemoglobin (%); HOMA-IR, homeostasis model assessment ((gluc mg/dl*insul U/ml)/405); Cholesterol (mg/dl); TG, triglycerides (mg/dl); LDL-c, low density lipoprotein-cholesterol (mg/dl); HDL-c, high density lipoprotein-cholesterol (mg/dl); CRP, c-reactive protein (mg/l); Adiponectin (ng/ml); Leptin (ng/ml); FASN, fatty acid synthase; PPARγ, peroxisome proliferator-activated receptor. Relationship between variables in control and hyperglycemic individuals was assessed by Student's *t *test.

Differences according to sex (data not shown) for clinical and laboratory data have shown that leptin and adiponectin levels were significantly higher in females (P < 0.01), the same as CRP (P < 0.05) and HDL-c (P < 0.01). No differences were found in the rest of variables between sexes.

**Figure 1:**
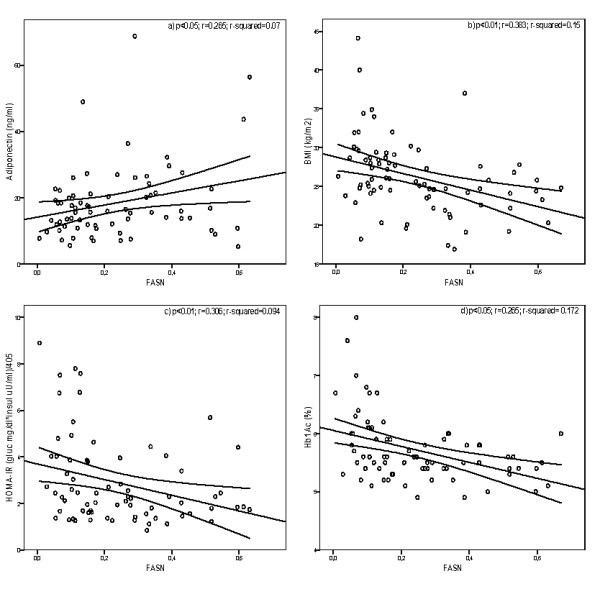
**Linear relationship between FASN expression and adiponectin (a), BMI (b), HOMA-IR (c) and HbA1c (d)**. Linear relationship was determined by Pearson's correlation coefficient test. 95% confidence interval.

The correlation between FASN expression and the different parameters that are associated with diabetes and obesity have shown the following results: there is a positive correlation of FASN expression with levels of adiponectin (P < 0.05; r = 0.265; Figure 1a) and HDL-c (P < 0.05; r = 0.276). BMI (P < 0.01; r = 0.383; Figure 1b), W-H (P < 0.05; r = 0.274), glucose (P < 0.01; r = 0.373), HOMA-IR (P < 0.01; r = 0.306; Figure 1c), HbA1c (P < 0.01; r = 0.415; Figure 1d) and TG (P < 0.01; r = 0.339) correlates inversely with FASN expression.

Multiple regression analysis (Table 3) found that FASN expression levels (as dependent variable) were related to values of HbA1c (P < 0.01) and BMI (P < 0.01) with a value of the model of R^2 ^= 0.385 and R^2 ^= 0.271 respectively. Variables that did not enter in the model were TG, HDL-c, HOMA-IR, adiponectin and PPARγ1 and PPARγ2 (data not shown).

**Table 3:** Multiple regression analysis

		Nonestandardized Coefficients		Standardized Coefficients	t	P	R^2^
			
Model		B	**Tip. Error**.	Beta			
1	(Constant)	0.718	0.120		5.980	0.000	0.271
		
	BMI	-0.019	0.004	-0.521	-4.182	0.000	

2	(Constant)	1.110	0.175		6.353	0.000	0.385
		
	BMI	-0.013	0.005	-0.371	-2.936	0.005	
		
	HbA1c	-0.093	0.032	-0.369	-2.913	0.006	

BMI, body mass index (Kg/m^2^); W-H, waist to hip ratio; Glucose (mg/dl); HbA1c, glycated haemoglobin (%); HOMA-IR, homeostasis model assessment ((gluc mg/dl*insul U/ml)/405); TG, triglycerides (mg/dl); LDL-c, low density lipoprotein-cholesterol (mg/dl); HDL-c, high density lipoprotein-cholesterol (mg/dl); Adiponectin (ng/ml); FASN, fatty acid synthase; PPARγ, peroxisome proliferator-activated receptor.

Dependent variable: FASN

Excluded variables: PPARγ1, γ2, Adiponectin, HOMA-IR, Glucose, TG, HDL-c, W-H

## Discussion

We investigated how FASN gene expression in human adipose tissue is related to carbohydrate metabolism dysfunctions and obesity. FASN gene expression was studied in adipose tissue using quantitative RT-PCR in samples of visceral adipose tissue from 87 volunteers who varied in terms of BMI, sex and metabolic parameters. We used correlation analysis to dissect whether and to what extent FASN mRNA expression is explained by the variability in anthropometric and metabolic parameters and we found an inverse correlation of FASN with Glucose, HOMA-IR, HbA1c, TG, BMI and W-H, while there was a positively correlation with adiponectin and HDL.

Feeding on simple carbohydrates substantially increases the activity of FASN, the central enzyme for de novo synthesis of long-chain saturated FAs [22]. FASN expression and activity are increased by insulin in cultured human adipocytes, suggesting that insulin sensitivity plays a role in their regulation and is essential in the uptake of glucose and conversion to TG. Insulin stimulates the transcription of lipogenic genes in rat hepatocytes and adipocytes, and this action has been confirmed in human adipocytes [23]. The results of the present study also demonstrate that adipose FASN gene expression is higher in normoglycemic individuals compared to those with hyperglycaemia, together with lower values of BMI, TG and obviously glucose, HOMA-IR, and HbA1c levels in normoglycemics. The relation between FASN and glycaemia is corroborated by multiple regression analysis where we have demonstrated the close relation of FASN expression with HbA1c. Due to the fact that HbA1c is image of medium values of glycaemia in the last three months, we took this value as representative of glycaemia state. This relation is of more importance when we take into account that what is being analyzed is a population with a wide range of BMI and metabolic parameters. Moreover, FASN is a variable that plays a role in body weight regulation and the development of obesity [13-15]. In this and previous studies, our laboratory has found that FASN relates inversely with obesity and this suggests that it could play a role in obesity-associated diabetes.

Our study design also allowed us to investigate the relationship between FASN mRNA expression and serum concentrations of adipocytokines (leptin and adiponectin). We found a correlation between FASN and serum concentrations of adiponectin. These adipocytokines are also BMI dependent in obesity while leptin increases, adiponectin decreases. According to sex we can also see that both are present in higher concentrations in women than in men. Leptin could directly suppress FASN mRNA expression in adipose tissue, since experimentally increased plasma leptin concentrations in rats resulted in a decrease of FASN mRNA levels in fat [24]. There are data supporting a suppressive action of leptin on FAS transcription [25]. Adiponectin is an exclusively adipocyte-derived hormone [26] with a key role in glucose and lipid metabolism in skeletal muscle and the liver, acting as an insulin sensitizer [27]. It is the only adipocytokine known to be down-regulated in obesity [28] and insulin resistance by decreasing TG content in muscle and liver [29]. Hypoadiponectinemia has been more closely related to the degree of insulin resistance and hyperinsulinemia than the degree of adiposity [28].

PPARγ has been implicated in almost all aspects of the cluster of human diseases designated as metabolic syndrome [6,7]. Because of this, it is a good candidate to study, crucial for whole-body insulin sensitivity [30] and adipogenesis [8]. The actions of PPARγ are mediated by two protein isoforms, the widely expressed PPARγ1 and the adipose tissue-restricted PPARγ2 [9]. PPARγ also transcriptionally regulates many genes involved in metabolism [12]. But we have found no significant changes in PPARγ1 and PPARγ2 expression levels related to carbohydrate metabolism or FASN expression levels. Our results do not support the relation of PPARγ with FASN and insulin sensitivity. On the other hand, PPARγ activation is also associated with potentially beneficial effects on the expression and secretion of adipocytokines [30] which protect nonadipose tissue against lipid overload. Increased TNFα, leptin, and resistin levels and decreased adiponectin expression in adipose tissues are associated with the development of insulin resistance and vice versa [28,30].

## Conclusions

Taken together, it has been demonstrated that FASN is a candidate gene for the pathophysiology of human obesity and type II diabetes and we corroborate this with the correlation of adipose FASN mRNA expression with several parameters related to obesity and diabetes.

## Competing interests

The authors declare that they have no competing interests.

## Authors' contributions

MDM drafted the manuscript, designed the study, participated in the genetic studies and in the analysis of biochemical variables, and performed the statistical analysis. FJO and MMG carried out the genetic studies. RB analyzed biochemical variables. RGH obtained the anthropometrical characteristics and the written consent of patients. JMFR participated in the conception and the coordination of the study. FJT carried out the conception, design and the coordination of the study, and helped with the statistical analysis. All authors read and approved the final manuscript.

## References

[B1] Klaus S Adipose tissue as a regulator of energy balance Curr Drug Targets 2004 5 241 250 10.2174/1389450043490523 15058310

[B2] Wajchenberg BL Subcutaneous and visceral adipose tissue: their relation to the metabolic syndrome Endocr Rev 2000 21 697 738 10.1210/er.21.6.697 11133069

[B3] Grundy SM Obesity, metabolic syndrome, and cardiovascular disease J Clin Endocrinol Metab 2004 89 2595 2600 10.1210/jc.2004-0372 15181029

[B4] Tsuchida A Yamauchi T Kadowaki T Nuclear Receptors as Targets for Drug Development: Molecular Mechanisms for Regulation of Obesity and Insulin Resistance by Peroxisome Proliferator-Activated Receptor, CREB-Binding Protein, and Adiponectin J Pharmacol Sci 2005 97 164 170 10.1254/jphs.FMJ04008X2 15725703

[B5] Frayn KN Adipose tissue as a buffer for dayly lipid flux Diabetologia 2002 45 1201 1210 10.1007/s00125-002-0873-y 12242452

[B6] Scott CL Diagnosis, prevention, and intervention for the metabolic syndrome Am J Cardiol 2003 92 35i 42i 10.1016/S0002-9149(03)00507-1 12867253

[B7] Ginsberg HN Treatment for patients with the metabolic syndrome Am J Cardiol 2003 91 29E 39E 10.1016/S0002-9149(02)03386-6 12679201

[B8] Rosen ED Spiegelman BM PPARγ: a nuclear regulator of metabolism, differentiation, and cell growth J Biol Chem 2001 276 37731 37734 10.1074/jbc.M106424200 11459852

[B9] Vidal-Puig AJ Considine RV Jimenez-Linan M Werman A Pories WJ Caro JF Flier JS Peroxisome proliferator-activated receptor gene expression in human tissues. Effects of obesity, weight loss, and regulation by insulin and glucocorticoids J Clin Invest 1997 99 2416 2422 10.1172/JCI119424 9153284 PMC508081

[B10] Cock TA Houten SM Auwerx J Peroxisome proliferator-activated receptor-γ: too much of a good thing causes harm EMBO reports 2004 5 Suppl 2 142 147 10.1038/sj.embor.7400082 14755307 PMC1298993

[B11] Barroso I Gurnell M Crowley VE Agostini M Schwabe JW Soos MA Maslen GL Williams TD Lewis H Schafer AJ Chatterjee VK O'Rahilly S Dominant negative mutations in human PPAR_ associated with severe insulin resistance, diabetes mellitus and hypertension Nature 1999 402 880 883 10622252 10.1038/47254

[B12] Fajas L Debril MB Auwerx J Peroxisome proliferator-activated receptor-gamma: From adipogenesis to carcinogenesis J Mol Endocrinol 2001 27 1 9 10.1677/jme.0.0270001 11463572

[B13] Mobbs CV Makimura H Block the FAS, lose the fat Nat Med 2002 8 335 336 10.1038/nm0402-335 11927935

[B14] Loftus TM Jaworsky DE Frehywot GL Townsend CA Ronnett GV Lane MD Kuhajda FP Reduced food intake and body weight in mice treated with fatty acid synthase inhibitors Science 2000 288 2379 2381 10.1126/science.288.5475.2379 10875926

[B15] Diraison F Dusserre E Vidal H Sothier M Beylot M Increased hepatic lipogenesis but decreased expression of lipogenic gene in adipose tissue in human obesity Am J Physiol Endocrinol Metab 2002 282 46 51 10.1152/ajpendo.2002.282.1.E46 11739082

[B16] Wakil S Fatty acid synthase, a proficient multifunctional enzyme Biochemistry 1989 28 4523 4530 10.1021/bi00437a001 2669958

[B17] Berndt J Kovacs P Ruschke K Klöting N Fasshauer M Schön MR Körner A Stumvoll M Blüher M Fatty acid synthase gene expression in human adipose tissue: association with obesity and type 2 diabetes Diabetologia 2007 50 1472 1480 10.1007/s00125-007-0689-x 17492427

[B18] Blüher M Michael MD Peroni OD Ueki K Carter N Kahn BB Kanh CR Adipose tissue selective insulin receptor knockout protects against obesity and obesity-related glucose intolerance Dev Cell 2002 3 25 38 10.1016/S1534-5807(02)00199-5 12110165

[B19] Blüher M Patti ME Gesta S Kahn BB Kahn CR Intrinsic heterogeneity in adipose tissue of fat-specific insulin receptor knock-out mice is associated with differences in patterns of gene expression J Biol Chem 2004 279 31891 31901 10.1074/jbc.M404569200 15131119

[B20] Turner SM Roy S Sul HS Neese RA Murphy EJ Samandi W Roohk DJ Hellerstein MK Dissociation between adipose tissue fluxes and lipogenic gene expression in ob/ob mice Am J Physiol Endocrinol Metab 2007 292 E1101 E1109 10.1152/ajpendo.00309.2005 17164440 PMC2895312

[B21] García-Fuentes E García-Almeida JM García-Arnés J Rivas-Marín J Gallego-Perales JL González-Jiménez B Cardona I García-Serrano S Garriga MJ Gonzalo M de Adana MS Soriguer F The cannabinoid CB1 receptor antagonist SR141716A (Rimonabant) enhances the metabolic benefits of long-term treatment with oleoylethanolamide in Zucker rats Neuropharmacology 2008 54 226 34 10.1016/j.neuropharm.2007.03.007 17467748

[B22] Hillgartner FB Salati LM Goodridge AG Physiological and molecular mechanisms involved in nutritional regulation of fatty acid synthesis Physiol Rev 1995 75 47 76 7831398 10.1152/physrev.1995.75.1.47

[B23] Wang Y Jones Voy B Urs S Kim S Soltani-Bejnood M Quigley N Heo YR Standridge M Andersen B Dhar M Joshi R Wortman P Taylor JW Chun J Leuze M Claycombe K Saxton AM Moustaid-Moussa N The human fatty acid synthase gene and de novo lipogenesis are coordinately regulated in human adipose tissue J Nutr 2004 134 1032 1038 15113941 10.1093/jn/134.5.1032

[B24] Nogalska A Sucajtys-Szulc E Swierczynski J Leptin decreases lipogenic enzyme gene expression through modification of SREBP-1c gene expression in white adipose tissue of aging rats Metabolism 2005 54 1041 1047 10.1016/j.metabol.2005.03.007 16092054

[B25] Bai Y Zhang S Kim K Lee J Kim R Obese gene expression alters the ability of 30A5 prreadipocytes to respond to lipogenic hormones J Biol Chem 1996 271 13939 13942 10.1074/jbc.271.24.13939 8663251

[B26] Maeda N Takahashi M Funahashi T Kihara S Nishizawa H Kishida K Nagaretani H Matsuda M Kouro R Ouchi N Kuriyama H Hotta K Nakamura T Shimomura I Matsuzawa Y PPARgamma ligands increase expression and plasma concentrations of adiponectin, an adipose-derived protein Diabetes 2001 50 2094 2099 10.2337/diabetes.50.9.2094 11522676

[B27] Chandran M Phillips SA Ciaraldi T Henry RR Adiponectin: more than just another fat cell hormone? Diabetes Care 2003 26 2442 2450 10.2337/diacare.26.8.2442 12882876

[B28] Weyer C Funahashi T Tanaka S Hotta K Matsuzawa Y Pratley RE Tataranni PA Hypoadiponectinemia in obesity and type 2 diabetes: Close association with insulin resistance and hyperinsulinemia J Clin Endocrinol Metab 2001 86 1930 1935 10.1210/jc.86.5.1930 11344187

[B29] Arita Y Kihara S Ouchi N Takahashi M Maeda K Miyagawa J Hotta K Shimomura I Nakamura T Miyaoka K Kuriyama H Nishida M Yamashita S Okubo K Matsubara K Muraguchi M Ohmoto Y Funahashi T Matsuzawa Y Paradoxical decrease of an adipose-specific protein, adiponectin, in obesity Biochem Biophys Res Commun 1999 257 79 83 10.1006/bbrc.1999.0255 10092513

[B30] Di Gregorio GB Yao-Borengasser A Rasouli N Varma V Lu T Miles LM Ranganathan G Peterson CA McGehee RE Kern PA Expression of CD68 and macrophage chemoattractant protein-1 genes in human adipose and muscle tissues: association with cytokine expression, insulin resistance, and reduction by pioglitazone Diabetes 2005 54 2305 2313 10.2337/diabetes.54.8.2305 16046295

